# Epigenetics: The Key to Future Diagnostics and Therapeutics of Lung Cancer

**DOI:** 10.7759/cureus.19770

**Published:** 2021-11-20

**Authors:** Gaurav Jha, Sabeen Azhar, Usman Rashid, Hasan Khalaf, Noor Alhalabi, Deepthi Ravindran, Rawaha Ahmad

**Affiliations:** 1 Neurology/Stroke Medicine, Barking Havering and Redbridge University Hospitals NHS Trust, Queen's Hospital, London, GBR; 2 Acute Medicine, Barking Havering and Redbridge University Hospitals NHS Trust, Queen's Hospital, London, GBR; 3 Stroke Medicine, Barking Havering and Redbridge University Hospitals NHS Trust, Queen's Hospital, London, GBR; 4 General Surgery, Barts Health NHS Trust, Whipps Cross Hospital, London, GBR; 5 Acute Medicine, Barking Havering and Redbridge University Hospitals NHS Trust, King George Hospital, London, GBR

**Keywords:** histone modifications, epigenetics, microrna, tumour biomarkers, dna methylation, non-small cell lung carcinoma (nsclc)

## Abstract

Lung cancer is still the major cause of cancer-related mortality around the globe. The interplay of permanent genetic and dynamic epigenetic changes leads to the onset and progression of lung cancer. The diagnosis is often made at an advanced stage when the prognosis is dismal and therapy choices are restricted. Epigenetic association with lung cancer has long been studied but with fewer success rates. Research is still progressing, and with an advanced understanding of human genomics, more and more information is being unveiled. In the last decade, epigenetics and particularly research on DNA methylation and histone modification have provided vital information to understand lung cancer pathogenesis better. As a result, stage-specific epigenetic modifications can be employed as strong and reliable tools for early lung cancer detection and patient prognosis monitoring. The information on epigenetic biomarkers for lung cancer is summarised in this review, which focuses on DNA methylation and histone modification, as well as its implications for early detection, diagnosis, prognostication, and treatments.

## Introduction and background

Epigenetics, initially defined by C.H. Waddington as 'the cause-and-effect connections between genes and their products that give rise to the phenotype', involves understanding chromatin structure and its impact on gene function [1]. However, because epigenetics has been linked to various biological processes, its meaning has changed through time.

Lung cancer is the most common cancer in both men and women, and it continues to be the leading cause of cancer-related deaths globally, with an expected 2.2 million new cases and 1.8 million deaths in 2020 [2]. The high death rate is due to the disease's great prevalence, as well as its terrible five-year survival rate of only 17% [2]. Approximately 85% of cases are non-small cell lung cancer (NSCLC) [3]. In the last decade, epigenetics and particularly research on deoxyribonucleic acid (DNA) methylation and histone modification have provided vital information to understand lung cancer pathogenesis better.

The interplay of genetic, epigenetic, and environmental variables leads to the initiation and development of lung cancer. DNA undergoes multiple processes during transcription, including DNA methylation and histone acylation, which are disrupted in lung cancer, the biggest cause of cancer-related mortality globally. The major factors in the epigenomics of lung cancer include gene silencing induced by hypermethylation, chromosomal instability produced by hypomethylation, and translation remodeling mediated by histone deacylation. After exposure to various environmental risk factors such as smoking, medications, and chronic inflammation, these modifications can occur in specific nuclear locations and chromosomal domains [4]. According to epidemiological research, cigarette smoking is responsible for 80-90% of lung cancers [5]. Despite the fact that the majority of lung cancer patients are smokers, only around 10% of lifetime smokers will acquire the illness [6]. This clearly suggests that vulnerability to lung cancer is caused by genetic and epigenetic factors [7].

Early detection of lung cancer can have a substantial impact on the disease prognosis; indeed, the survival rate can skyrocket. Many imaging and cytology-based procedures have been used in the endeavor to enhance early detection; however, none of them has shown to be particularly effective, either due to low sensitivity or the exorbitant expense they impose on public health systems [8,9]. The most effective strategy to develop novel diagnostic and treatment tools is to understand the molecular pathways within lung cancer and focus on their molecular heterogeneity. Recent advancements in lung cancer epigenetics represent a significant step forward in the development of new biomarkers.

## Review

Tumour biomarkers

**Figure 1:**
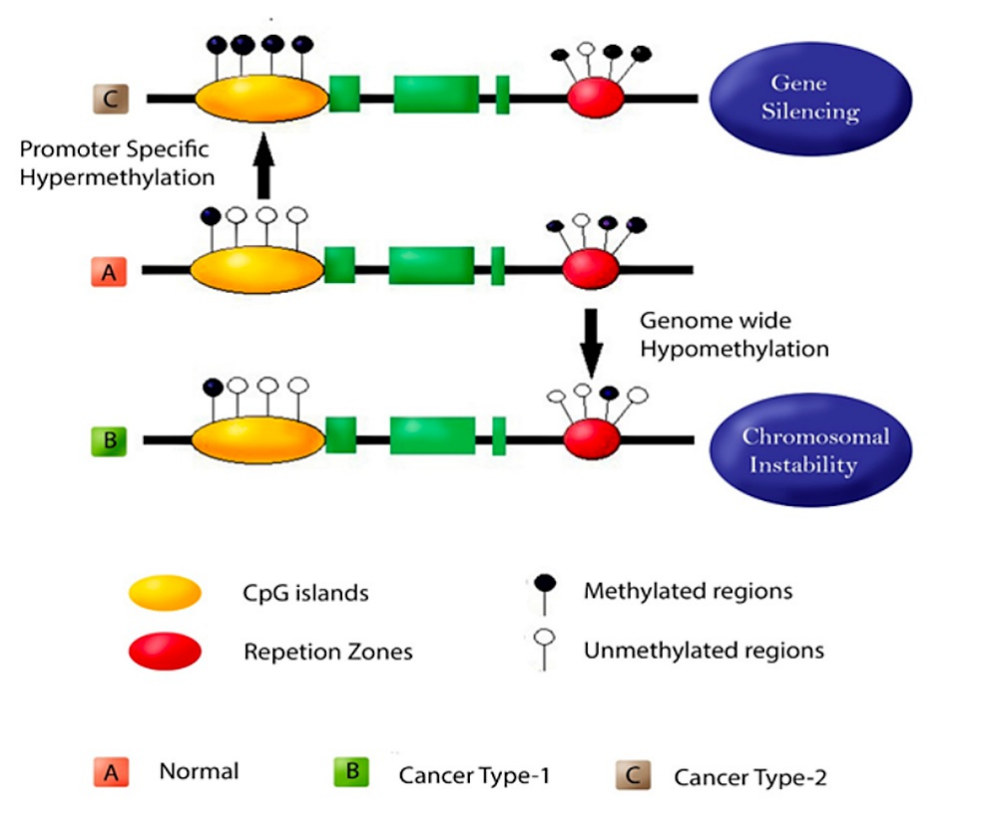
Schematic representation of epigenetic mechanisms

Biomarkers, according to the National Cancer Institute, are biological molecules present in the blood, other body fluids, or tissues that signal whether a process, condition, or illness is normal or abnormal. Cancer biomarkers have improved the validity of risk assessment and early detection tactics and provided insight into disease progression and prognosis, all of which have proven crucial in developing targeted individualised cancer therapy. The discovery of new biomarkers to help in outcome prediction and tumour response is critical for optimising therapy efficacy and avoiding over-or under-treatment of lung cancer patients. Epigenetic biomarkers, particularly DNA methylation, histone modifications, and micro ribonucleic acid (RNA) expression, have unique features that make them promising prognostic indicators (Figure 1). They are not only valuable for the early diagnosis of cancer but also provide important prognostic information, predicting how aggressive the disease process would be and differentiating the tumour's outcome [10].

Epigenetics refers to the second layer of information encoded on the genome that governs genomic function and activity. Epigenetic alteration offers excellent potential as a cancer biomarker since it can be used to analyse specific, reversible changes in disease-relevant genes and monitor important events in carcinogenesis. Another critical application is predicting therapeutic response; predictive biomarkers assess the likely effectiveness of a specific treatment. Tumour biomarkers provide essential clinical information in selecting suitable treatment, leading to customised cancer therapy [11].

DNA methylation as a tumour biomarker

DNA methylation changes in tumorigenesis have recently emerged as a viable prospective biomarker for cancer patients' early identification, prognosis, therapy response prediction, and recurrence monitoring.

The chemical covalent alteration of cytosine in DNA is known as methylation, where a methyl group (CH3) is attached to the 5-C of the cytosine residue called 5’-cytosine-phosphate-guanine-3' (CpG) dinucleotides by DNA methyltransferases (DNMTs). The distribution of CpG dinucleotides in the genome is uneven, with CpG dinucleotides clustered in small areas known as CpG islands (CGIs). A methyl group is transferred from S-adenosyl-L-methionine to the 5'-cytosine carbon of CpG islands by three active DNMTs (DNMT1, DNMT3a, and DNMT3b) [12-14]. Promoter-specific hypermethylation of the 'unmethylated' CpG island is the striking mechanism of epigenetic associated cancer modification.

Epigenetic changes can be broadly characterised as focal hypermethylation and global hypomethylation exhibiting an abnormal methylation signature of tumour suppressor genes (TSGs) [15]. Hypermethylation can silence essential tumour suppressors or regulatory areas in the genome, resulting in dysregulation of cell proliferation or altered responsiveness to cancer therapy [16]. These epigenetic processes can work with known driver mutations to promote cancer development or evolution [17]. Many genes are hypermethylated in lung cancer. Methylation of cyclin-dependent kinase-2 (cdk2A), adenomatous polyposis coli (APC), Cadherin-13 (CDH13), p16 (also known as cyclin-dependent kinase inhibitor 2A, CDKN2A), and Ras association domain family protein1 isoform A (RASSF1A), for example, has been linked to recurrence after surgical resection of stage I NSCLC, regardless of histological background, gender, or smoking history [18]. Another study found that p16 methylation and subsequent p16 expression reduction were linked to worse survival after early-stage NSCLC resection [19].

DNA hypermethylation can be detected in lung cancer patients' bronchoscopic washings/brushings, aspirates from sputum, and blood (plasma and serum), all of which are less invasive and less painful than a tumour biopsy. These technologies might potentially help detect extremely early lung tumours or newly recurrent malignancies that would be undetected by conventional methods in the future [16]. DNA methylation has been found as a potential therapeutic via DNMT enzyme inhibition, in addition to its usefulness as a predictive and prognostic biomarker. The two main DNMTs inhibitors, 5-azacitidine, and decitabine, have already been extensively studied in the clinic [12]. 5-azacitidine is incorporated into DNA and RNA after phosphorylation, followed by the covalent trapping of DNMTs to the DNA, resulting in proteasomal degradation and a reduction in global DNA methylation. The direct cytotoxicity of these medications is produced by DNA damage and reduced DNA synthesis induced by DNA-DNMT adducts when administered at higher doses. Unlike 5'-azacitidine, decitabine is not incorporated into RNA and is only selective for DNA [14].

Genomic hypomethylation or a loss of methylation occurs throughout the whole genome, which generally occurs later in lung cancer development. Gene-specific hypermethylation, on the other hand, may occur early in cancer formation [20]. However, since hypomethylation has been associated with the progression of NSCLC from average to lung cancer, there is still no clear consensus on time. Hypomethylation is a carcinogenic condition that occurs in repetitive locations. Regardless, extensive hypomethylation has been linked to genomic instability in NSCLC, leading to oncogene activation, missegregation of chromosomes during cell division, unwanted activation of transposable elements within the genome, and imprinting loss. In lung cancer, hypomethylation is more common in long terminal repeats (LTR), nuclear components, subtelomeric regions (loss of methylation is much less common at non-repetitive sequences), and segmental duplicates [21]. Hypomethylation occurs predominantly in repetitive areas and has been demonstrated to be a carcinogenic process.

Histone modifications

It is well known that histone post-translation modification (PTM) regulates a wide range of crucial biological processes, most notably chromatin modification that promotes or inhibits the expression or repression of target genes. The majority of study has focused on acetylation, methylation, and phosphorylation. However, histone deacetylation, or the removal of acetyl groups by histone deacetylases (HDACs), plays a vital role in tumorigenesis. Histone acetyltransferases (HATs) are enzymes that add an acetyl group to the lysine residue on the N-terminal tails of histones. This mechanism is controlled by DNA methylation, which results in the accumulation of repressor proteins in CpG islands and post-translational modification of histones. Abnormal gene expression is induced by an imbalance between acetylation and deacetylation, which leads to cell proliferation, DNA replication, mitosis, and carcinogenesis. HDAC inhibitors are gaining popularity as new anti-cancer drugs due to their ability to kill cancer cells by autophagy, apoptosis, cellular necrosis, cell cycle arrest, production of reactive oxygen species (ROS), reduction of tumour angiogenesis, and immunomodulatory effects. They lower the total tumour cell apoptotic threshold by stimulating both death-receptor and intrinsic mitochondrial mechanisms. They increase the expression of pro-apoptotic genes involved in the death receptor pathway (tumor necrosis factor-related apoptosis-inducing ligand, TRAIL and death receptor, DR5) as well as the intrinsic apoptotic pathway like Bax, Bak, and apoptotic peptidase activating factor 1 (APAF1) while decreasing the expression of pro-survival genes (BCL-2 and XIAP). They also initiate the intrinsic apoptotic pathway by selectively activating or inducing BH3-only proteins [22,23]. HDACIs stimulate the immune system by boosting the synthesis of MHC class I and II proteins, along with co-stimulatory/adhesion molecules such CD80, CD86, intracellular adhesion molecule-1 (ICAM-1,28), and human leukocyte antigen HLA-DR, HLA-ABC. Angiogenesis, a vital element in tumour invasion and metastasis, can also be prevented by HDACIs [22]. Lung cancer cells show aberrant histone H4 modification patterns, such as hyperacetylation of H4K5/H4K8, hypoacetylation of H4K12/H4K16, and a lack of trimethylation of H4K20. Their findings underline the significance of histone H4 changes and H4K20me3 as a potential biomarker in order to diagnose and treat lung cancer as early as possible. Furthermore, the differently expressed gene pattern of HATs and HDACs in tumour samples versus regular counterparts may have therapeutic implications, such as early tumour identification, prognosis, and the direction of epigenetic-targeted treatment [24]. In HDI-treated NSCLC cells, TNF-receptor-1 mRNA, surface protein expression, and protein levels were reduced, indicating that TNF therapy inhibited NF-B nuclear translocation and DNA binding. As a result, HDIs may aid tumour therapy by reducing tumour cell responsiveness to TNF-mediated NF-B pathway activation [25].

Early detection and diagnosis

Lung cancer mortality could be considerably decreased if the disease is detected earlier. However, only around 15% of lung tumours are localised at the time of diagnosis, with the vast majority presenting at an advanced stage [26]. A diagnostic molecular biomarker should discriminate between neoplastic transformation and preneoplasia to represent the early stages of cancer development. Several hypotheses and speculations about epigenomics-related cancer continue to emerge and remain unproven. Hypermethylation of the promoter can be a precursor to lung cancer. As a result, as demonstrated by CDKN2A and methylguanine-methyltransferase (MGMT) promoter methylation, it might help identify the disease up to three years before a diagnosis. The p16 promoter was perhaps the first to be shown to be hypermethylated early in lung carcinogenesis, and subsequent studies found abnormal hypermethylation of several gene promoters, including RARb, APC, RASSF1, and MGMT [27,28]. 

Prognosis and prediction

Tumour characteristics, including pathological subtype, nodal invasion, and metastasis, have traditionally been used to predict illness outcomes. Aside from these well-established prognostic factors, aberrant DNA methylation and microRNA expression have been widely researched and published in the literature due to the distinct properties that make them well-suited as prospective prognosis markers. The development of novel biomarkers to aid in predicting outcome and tumour response is crucial for improving therapeutic efficacy and preventing over-or under-treatment of lung cancer patients. TSG hypermethylation is essentially related to poor outcomes. Zhang et al. utilized methylation-explicit polymerase chain reaction (PCR) to explore the methylation status of 15 genes in 64 matched NSCLC and encompassing normal tissues. The analysts found that patients with at least four methylated genes simultaneously had a worse two-year progression-free survival [29]. Lung cancer survival has been connected to several global histone modifications specifically, lower levels of H3K4diMe have now been linked to a worse outcome. Furthermore, the combination of several histone modifications (H3K4me2, H3K9ac, and H2AK5ac) has been found to predict survival significantly, but H4K20me3 down-regulation has been linked to lower survival in patients with stage I lung adenocarcinoma [30].

MiRNA methylation as a new biomarker in lung cancer

MiRNAs are hypothesised to be novel epigenetic modifiers of gene expression that play critical roles in a wide range of biological processes. They are commonly released from tissues as exosomes or microvesicles after apoptosis and found in the bloodstream. MicroRNA levels in the blood have been connected to prognosis. Several studies have discovered a relationship between microRNA activity in blood or plasma and lung cancer outcomes [31]. Although circulating DNA methylation may be helpful in diagnosis and prediction, it is crucial to differentiate free-circulating DNA from leukocytic DNA since DNA methylation marks are intricately tied to cellular differentiation and differ by cell type [32]. Hypermethylation of miR-124a modulates Rb phosphorylation and CDK6 activation in lung adenocarcinomas. The miRNA-29 family, on the other hand, may restore aberrant methylation in lung cancer by targeting the 3'-untranslated region of DNMT3A and DNMT3B, which are typically overexpressed in lung cancer and are associated with a poor prognosis. When MiR-29 is overexpressed, it can decrease lung carcinogenesis by normalising abnormal methylation in NSCLC by triggering re-expression of methylated silenced TSGs [33]. Researchers will build more comprehensive tumour diagnostics if they can establish a relationship between DNA methylation and miRNAs. Changes in miRs are rarely used clinically at the moment, and creating reliable miR panels for clinical application in lung cancer diagnosis, prognosis, and treatment will require several years [16].

## Conclusions

Epigenetics is the promising frontier of science entailed by copious reinforcing signals, embracing DNA methylation, micro RNAs, histone modifications, and chromatin remodelling. Epigenetics is essential in the aetiology of lung cancer and can be helpful as diagnostic and prognostic biomarkers. Large multicenter consortia are unavoidable to attain the statistical power needed for clinical biomarker validation and capitalise on DNA methylation's enormous promise to offer precise and sensitive diagnostic tools for early detection of lung cancer. The subject of DNA methylation biomarking is quickly growing, with promising discoveries that might have enormous implications for cancer patients in the future. Expanding our understanding of how epigenetic alterations contribute to lung cancer genesis and how they are being translated into clinically valuable markers and therapeutic targets will allow us to manage lung cancer effectively and, as a result, reduce the devastating global burden of this disease.
